# LUCAT1 promotes stemness in head and neck squamous cell carcinoma by sponging miR-128

**DOI:** 10.3389/fonc.2026.1795813

**Published:** 2026-06-03

**Authors:** Yifan Wen, Shuo Liu, Yaqing Mao, Ruifeng Li, Hui Yuh Soh, Yao Yu, Wen-bo Zhang, Lingfei Jia, Xin Peng

**Affiliations:** 1 Department of Oral and Maxillofacial Surgery, Peking University School and Hospital of Stomatology, Beijing, China; 2 National Center of Stomatology & National Clinical Research Center for Oral Diseases & National Engineering Laboratory for Digital and Material Technology of Stomatology, Beijing, China; 3 Department of Oral and Maxillofacial Surgery, Faculty of Dentistry, Universiti Kebangsaan Malaysia, Kuala Lumpur, Malaysia

**Keywords:** cancer stem cell (CSC), head and neck squamous cell carcinoma (HNSCC), long non-coding RNA (lncRNA), LUCAT1, microRNA, miR-128

## Abstract

Head and neck squamous cell carcinoma (HNSCC) progression is well documented to be predominantly driven by a small subset of cancer stem cells (CSCs). These CSCs are implicated in recurrence, metastasis, dormancy, and therapy resistance. Therefore, targeting cancer stem cells has inspired the design of innovative treatment strategies. This study demonstrates that knockdown of the long non-coding RNA (lncRNA) LUCAT1 markedly reduces cancer cell stemness. This effect was confirmed across several experimental assays, including sphere-forming capacity and *in vitro* limiting dilution assays. Consequently, LUCAT1 knockdown potently suppresses HNSCC progression. Mechanistically, LUCAT1 functions as a competing endogenous RNA (ceRNA) to sponge miR-128, thereby relieving the post-transcriptional repression of downstream targets and promoting oncogenesis. Functional rescue assays show that restoration of miR-128 significantly reverses the antitumor efficacy of LUCAT1 knockdown on HNSCC cell. Moreover, miR-128 also attenuates the antitumor efficacy of LUCAT1 knockdown in both subcutaneous xenograft models and lymph node metastasis models *in vivo*. In agreement with previous results, BMI1 was identified as a direct and functionally relevant target of miR-128 in HNSCC cells. Collectively, these findings show that LUCAT1 constitutes a key regulatory component within the cancer stemness regulatory network of HNSCC, and underscores the function of the LUCAT1/miR-128 axis in sustaining stem cell-like traits and driving HNSCC malignant progression.

## 1 Introduction

Head and neck squamous cell carcinoma (HNSCC) is the sixth most prevalent cancer worldwide, and its incidence continues to rise (1). Even with the progress in multimodality therapies, the five-year survival rate for HNSCC remains largely unchanged, and up to 50% of advanced cases experience either recurrence or metastasis (1, 2). A growing body of research indicates that cancer stem cells (CSCs) are the key driver of suboptimal treatment response and unfavorable clinical outcomes (3).

Previous studies have mostly focused on coding RNAs; beyond these, non-coding RNAs also serve as vital regulators of tumor stemness (3–5). The long non-coding RNA (lncRNA) LUCAT1 has been widely reported to promote the progression of various solid tumors, including lung, ovarian, and liver cancers. However, its functional relevance and mechanistic targets in HNSCC remain unexplored, particularly regarding the maintenance of cancer stem cells (6).

LUCAT1 interacts with diverse biomolecules, such as proteins, DNA, and other RNA transcripts that regulate oncogenic pathways (7). Escalating focus has been directed towards the role of competitive endogenous RNA (ceRNA) networks in cancer progression in recent years, within which microRNAs (miRNAs) act as core regulatory elements (8). To date, numerous miRNAs have been reported to serve as intermediate regulators or prognostic predictors in HNSCC (9–12). This highlights that targeting LUCAT1/miR–128/BMI1 axis may represent a potential therapeutic target for HNSCC, although its prognostic value and clinical relevance need further validation in patient cohorts.

Here, we confirmed LUCAT1’s indispensable role in HNSCC initiation, malignant progression, and the maintenance of cancer stem cell properties. Intriguingly, our results reveal that LUCAT1 exerts its regulatory effects on cancer stemness and oncogenic functions specifically through targeting miR-128, which acts as a key downstream mediator in the LUCAT1-centered ceRNA network. This regulatory cascade was validated across multiple HNSCC cell lines and xenograft models, and the efficacy of RNA-based intervention strategies targeting this pathway further underscores its translational value for HNSCC therapy.

## 2 Materials and methods

### 2.1 Cell culture and genetic manipulation

The human HNSCC cell lines HN6 and CAL27 were purchased from the American Type Culture Collection (ATCC, Manassas, VA, USA). Cells were maintained in Dulbecco’s Modified Eagle’s Medium (DMEM) supplemented with 100 μg/mL streptomycin, 100 U/mL penicillin, and 10% fetal bovine serum (FBS), and incubated at 37 °C in a humidified atmosphere containing 5% CO_2_.

For gene manipulation assays, adherent cells grown to 60% confluence underwent transfection with either negative control siRNA (siNC), two LUCAT1-targeting siRNAs (siLUCAT1-1, siLUCAT1-2), a miR-128 antagomir, or LUCAT1 overexpression (LUCAT1-OE) plasmid (Integrated Biotech Solutions Co., Ltd, Shanghai, China). Transfections were performed with JetPrime transfection reagent (Polyplus Transfection). All sequences were provided in **Supplementary Table 1**. Transfection efficiency was confirmed using quantitative real-time PCR (qRT-PCR) at 48 hours post-transfection. For subsequent LUCAT1 knockdown (KD) experiments, siLUCAT1-2—which exhibited superior knockdown efficiency—was selected for all functional assays.

### 2.2 Lentiviral transduction

LUCAT1 KD lentiviruses (shLUCAT1) and scramble control lentiviral vectors were produced through co-transfection of HEK293T cells with VSVG, REV, and Gag/Pol packaging plasmids (Integrated Biotech Solutions Co., Ltd). At 72 hours post-transfection, viral supernatants were harvested to infect target HNSCC cells with 8 μg/mL polybrene (Sigma-Aldrich, Shanghai, China), followed by puromycin selection (2 μg/mL). Prior to subsequent experiments, the knockdown efficiency of shLUCAT1 was validated via qRT-PCR.

### 2.3 Cell proliferation, migration, and invasion assay

Proliferation assay: Transfected cells (2×10^3^ per well) were cultured in 96-well plates under standard conditions. Cellular viability was subsequently assessed using the Cell Counting Kit-8 (CCK-8; Beyotime, Jiangsu, China).

Wound healing assay: Confluent monolayers in 6-well plates were scratched uniformly with a sterile 1-mL pipette tip. After gentle washing with phosphate-buffered saline (PBS) to remove dislodged cells, wound closure was observed via light microscopy. Images were taken at 24 hours for HN6 cells and 36 hours for CAL27 cells post-scratching. Image analysis was performed with ImageJ (v2.14; NIH, USA), and the percentage of wound closure was calculated using the formula: [(Initial wound width – Wound width at time point)]/(Initial wound width) × 100%.

Transwell migration and invasion assays: Transfected cells (1×10^5^ cells per well) were resuspended in serum-free DMEM and plated into the upper chamber of a Transwell insert (8 μm pore size, Corning, NY, USA). The lower compartment contained DMEM with 20% FBS as a chemoattractant. For invasion assessment, the membrane was pre-coated with Matrigel (Corning) before cell seeding, while no coating was used for migration assay. Following incubation under standard cell culture conditions, cells on the underside of the membrane were fixed with 4% paraformaldehyde (Solarbio, Beijing, China), stained with 0.1% crystal violet (Solarbio), and quantified using ImageJ software (v2.14; NIH). Five random fields were selected and analyzed per chamber.

### 2.4 qRT-PCR

Total RNA was extracted from transfected cells with TRIzol reagent (Invitrogen, Waltham, MA, USA). For reverse transcription (RT) reaction, 500 ng of mRNA was converted to complementary DNA (cDNA) using a commercial RT kit (Takara, Beijing, China) with random hexamer primers, while 1 μg of miRNA was reverse-transcribed with gene-specific stem-loop primers. Subsequently, quantitative real-time PCR was then carried out employing SYBR Green Master Mix (Roche, Basel, Switzerland). Target gene expression was normalized to the respective internal reference genes: GAPDH for mRNA and U6 small nuclear RNA (snRNA) for miRNA. Three independent biological replicates were performed for each assay. Primer sequence used in this study are provided in **Supplementary Table 2**.

### 2.5 Western blot

Cell lysis was performed using RIPA buffer (Solarbio) containing protease and phosphatase inhibitor mixtures (Huaxingbio, Chaohu, China). Protein separation was achieved via SDS-PAGE, followed by electrotransfer onto PVDF membranes. After blocking for one hour at ambient temperature with 10% non-fat dry milk, membranes were incubated overnight at 4 °C with primary antibodies: anti-BMI1 (1:1000, Cell Signaling Technology, Cat# 6964) and anti-GAPDH (1:1000, Cell Signaling Technology, Cat# 5174). Subsequently, membranes were treated with species-matched secondary antibodies for one hour at room temperature. Signal visualization was performed using a Clarity Western ECL detection kit (Invitrogen).

### 2.6 CSCs isolation and flow cytometry

CSCs with high aldehyde dehydrogenase (ALDH) activity were isolated from HN6 cell populations using the ALDEFLUOR assay kit (Stemcell Technologies, Vancouver, Canada). Briefly, single-cell suspensions were treated with the ALDEFLUOR substrate, with a parallel control group treated with the ALDH inhibitor diethylaminobenzaldehyde (DEAB) to establish gating parameters. The ALDH^+^ (ALDHhigh) cell population was subsequently identified and sorted via fluorescence-activated cell sorting (FACS). Data acquisition and analysis were conducted using FlowJo software (v10.9; Treestar Inc., Ashland, USA). The sorted ALDH^+^ CSCs were harvested and used for subsequent downstream functional assays.

### 2.7 Tumorsphere formation assay

The FACS-sorted cells were plated in ultralow attachment plates and maintained under the condition as previously described (13). After a ten-day incubation period, the number of spheroids exceeding 70 μm in diameter were quantified using an optical microscope (Olympus, Tokyo, Japan).

### 2.8 Extreme Limiting-dilution assay

HN6-derived ALDHhigh CSC-like cells were transduced with lentiviruses expressing either shNC or shLUCAT1 and rapidly selected with puromycin. Cells were diluted in a series of concentrations, combined with an equal volume of Matrigel, and administered subcutaneously to nude mice. Tumor formation was tracked over four weeks and assessed via the Extreme Limiting Dilution Analysis (ELDA) software (http://bioinf.wehi.edu.au/software/elda/). Tumor volume was calculated as (L × W²)/2 as described.

### 2.9 Animal model and *in vivo* procedures

Subcutaneous dorsal cell line-derived xenograft model: To assess tumor growth, HN6 cells (5 × 10^6^ cells/mouse) were resuspended in 100 μL of a 1:1 mixture of Matrigel (Corning) and serum-free DMEM. The cell suspension was injected into the dorsal flank of 6-8-week-old female BALB/c-nude mice (SiPeiFu Biotechnology Co., Ltd., Beijing, China). Three weeks after tumor implantation, mice were randomly allocated into four experimental groups (five mice per group). Treatments were administered twice weekly via multi−point intratumoral injection as follows: (1) negative control (NC); (2) LUCAT1 antisense oligonucleotide (ASO, 5 nmol/mouse; Integrated Biotech Solutions Co., Ltd., Shanghai, China); (3) miR-128 antagomir (5 nmol, Integrated Biotech Solutions Co., Ltd); (4) combination of LUCAT1 ASO and miR-128 antagomir. Tumor dimensions were recorded every three days with a digital caliper, and tumor volume was computed using the formula V = 0.5 × L × W² (L = length, W = width). At 5 weeks post-treatment initiation, mice were euthanized, and the tumors were surgically removed and weighed. The sequences of ASO used are listed in **Supplementary Table 1**.

Orthotopic xenograft model: To assess local tumor progression and lymph node metastasis, FACS-sorted ALDH^+^ CSC-like cells derived from HN6 cells—transduced with either scramble control lentivirus (shNC) or LUCAT1 KD lentivirus (shLUCAT1)—were sublingually inoculated into 6–8-week-old female BALB/c nude mice (SiPeiFu Biotechnology Co., Ltd.) at a density of 2 × 10^6^ cells/mouse. For *in vivo* rescue experiments, each group of tumor-bearing mice was randomly subdivided into two subgroups. These subgroups received intra-tumoral injections administered at multiple points within the tumor twice per week, consisting of either a miR-128 antagomir or a negative control antagomir (5 nmol/mouse; Integrated Biotech Solutions Co., Ltd.). Mice bearing orthotopic HNSCC tumors were monitored for 4 weeks and then euthanized. Primary tongue tumors and cervical lymph nodes were harvested for subsequent histological and molecular analyses. All animal experimental procedures received approval from the Animal Ethics Committee of Peking University and were performed following the institution’s guidelines for laboratory animal care and use.

### 2.10 Immunohistochemistry

Immunohistochemistry (IHC) was conducted following a previously published protocol (14). Briefly, 4-μm paraffin sections were incubated overnight at 4 °C with an anti-PCK primary antibody (1:200, Santa Cruz Biotechnology, Santa Cruz, CA, USA), followed by a horseradish peroxidase-conjugated secondary antibody (Zhongshan Golden Bridge) and DAB visualization. Nuclei were counterstained with hematoxylin. Images were acquired using an Olympus microscope, and a minimum of three sections were assessed per sample.

### 2.11 Nuclear and cytoplasmic fraction isolation

Cellular RNA was separated into nuclear and cytoplasmic fractions using a Nuclear and Cytoplasmic Protein Extraction Kit (Beyotime Biotechnology Co., Ltd., Shanghai, China). In this procedure, cells were lysed in ice-cold fractionation buffer, and nuclei were pelleted by low−speed centrifugation. Total RNA was extracted from both the nuclear pellet and cytoplasmic supernatant fractions using TRIzol reagent (consistent with the aforementioned protocol). The efficiency and specificity of subcellular fractionation were validated via qRT-PCR, using MALAT1 (a nuclear RNA control) and GAPDH (a cytoplasmic RNA control) as reference genes. Primer sequence used in this study are provided in **Supplementary Table 2**.

### 2.12 Luciferase reporter assays

The potential miRNAs binding sites on LUCAT1 were identified using RNA22 online prediction tool (https://cm.jefferson.edu/rna22/Interactive/). The wild-type or mutant sequences of LUCAT1 and BMI1 harboring the predicted target sites were inserted into the Psicheck dual-luciferase vector (Integrated Biotech Solutions Co., Ltd), and the resultant Psicheck -LUCAT1-wt/mut reporters and Psicheck -BMI1-wt/mut reporters were co-transfected with miR-128 mimics or negative control mimics into HN6 and CAL27 cells using JetPRIME reagent (Polyplus Transfection). Luciferase activities were quantified through the Dual-Glo Luciferase Assay System Kit (Beyotime). The sequences were provided in **Supplementary Table 1**.

### 2.13 Statistical analysis

Statistical analyses were conducted with GraphPad Prism 10.5.0. *In vitro* assays were conducted with a minimum of three biologically independent replicates, while *in vivo* studies utilized at least five animals per group for biological replication. Continuous variables are presented as mean ± SD following normality assessment with the Shapiro-Wilk test (n < 50), while categorical variables are presented as frequency (percentage). For statistical comparisons, Student’s t−test was applied for analyses involving two groups, while one−way ANOVA followed by Tukey’s *post-hoc* test was used for comparisons among multiple groups, provided the data followed a normal distribution. A two−tailed p−value of less than 0.05 was regarded as statistically significant.

## 3 Results

### 3.1 LUCAT1 knockdown suppresses HNSCC proliferation, migration, and invasion

**Figure 1:**
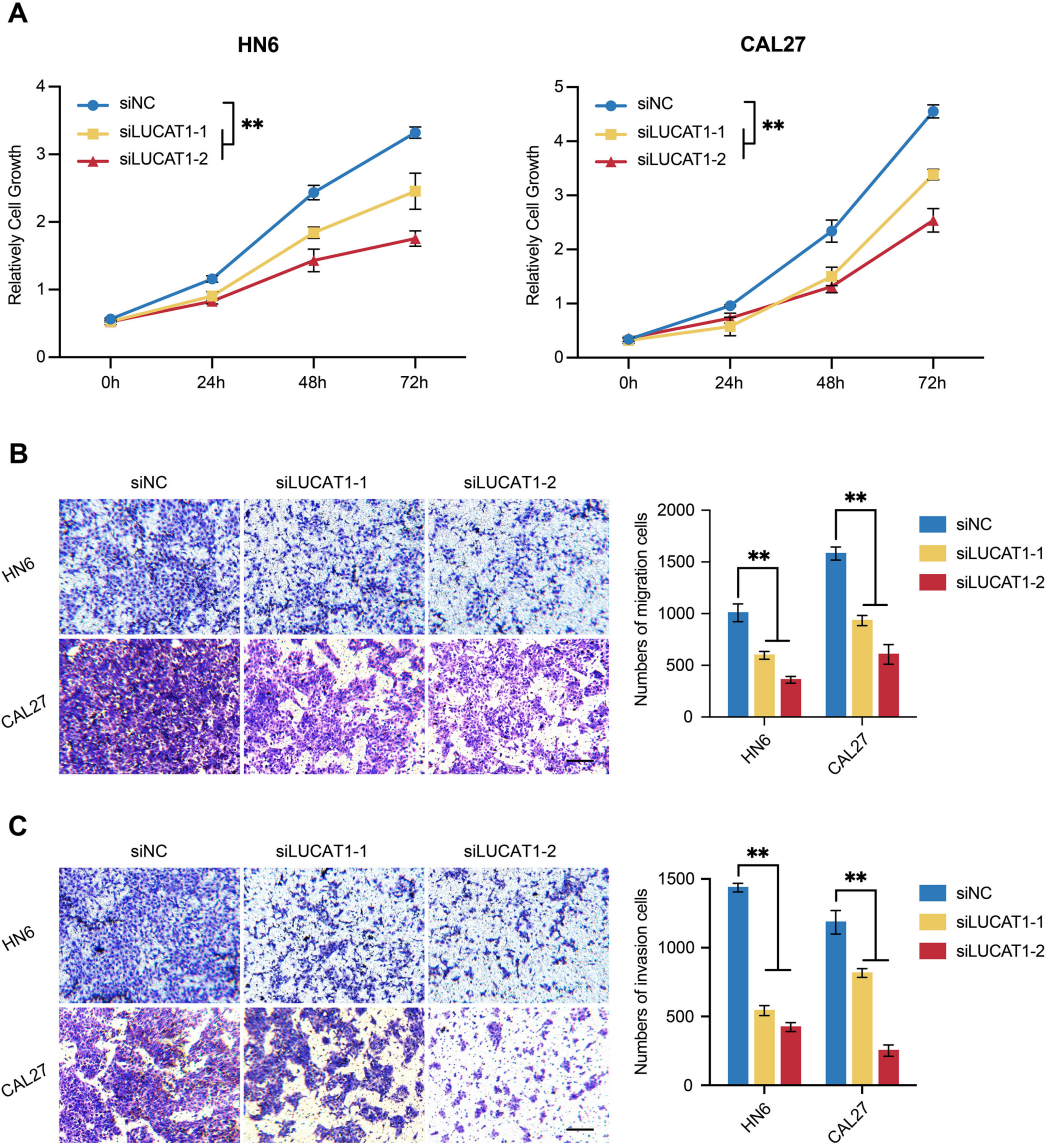
The biological effects of LUCAT1 in HNSCC. **(A)** Effect of LUCAT1 knockdown (KD) with two independent siRNAs (siLUCAT1-1, siLUCAT1-2) or control siRNA (siNC) on proliferation of HN6 and CAL27 cells. Mean ± SD are shown. **p < 0.01, unpaired Student’s t-test. **(B)** Transwell migration assays showing that LUCAT1 KD inhibits HN6 and CAL27 cells migration. Scale bar: 200 μm. Mean ± SD are shown. **p < 0.01, unpaired Student’s t-test. **(C)** Transwell invasion assays showing that LUCAT1 KD inhibits HN6 and CAL27 cells invasion. Scale bar: 200 μm. Mean ± SD are shown. **p < 0.01, unpaired Student’s t-test.

To elucidate LUCAT1’s oncogenic function in HNSCC and assess its potential contribution to tumor progression, HNSCC cell lines (HN6 and CAL27) were used for initial *in vitro* functional assays. LUCAT1 KD was mediated by small interfering RNA (siRNA), and efficient silencing of LUCAT1 expression was confirmed via qRT-PCR (**Supplementary Figure 1A**). CCK-8 proliferation assays indicated that knockdown of LUCAT1 inhibited the proliferation of HN6 and CAL27 cells (**Figure 1A**). Moreover, LUCAT1 KD markedly impaired the migratory capacity (**Figure 1B**) and invasive potential (**Figure 1C**) of HNSCC cells.

### 3.2 LUCAT1 knockdown inhibits tumorigenic potential of CSCs in HNSCC

**Figure 2:**
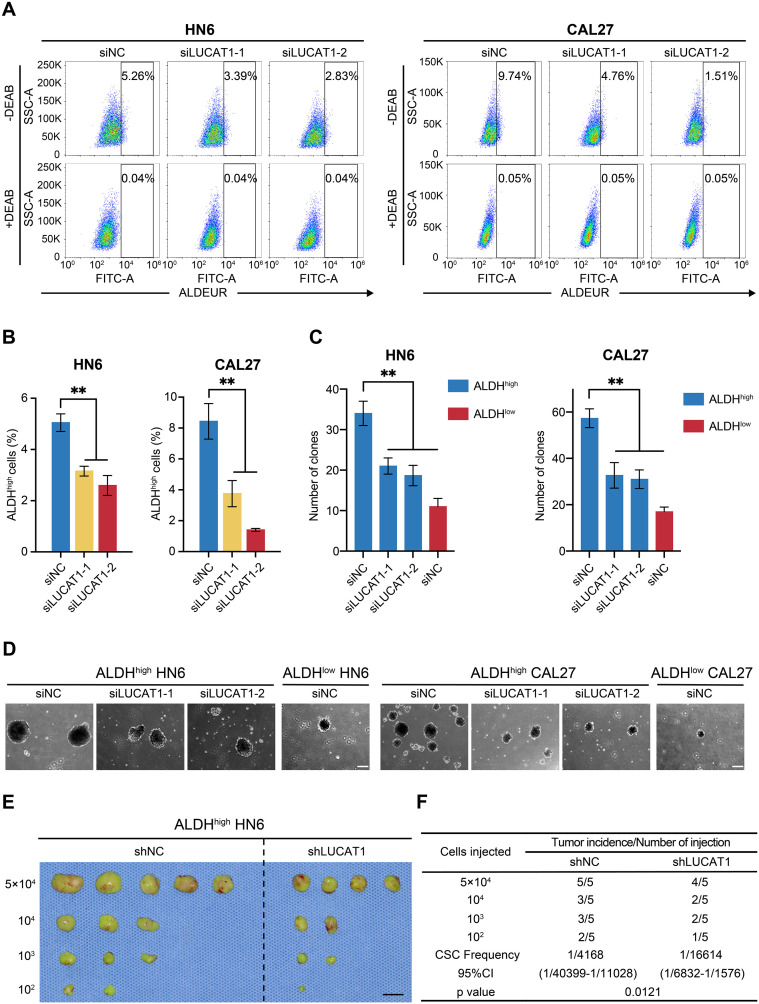
LUCAT1 down-regulation inhibits HNSCC stemness signature. **(A)** Representative FACS plots of ALDH^+^ (ALDH^high^) cells in HN6 and CAL27 cells with LUCAT1 KD. **(B)** Flow cytometry analysis showing that LUCAT1 KD reduces the proportion of CSCs in HN6 and CAL27 cells. Mean ± SD are shown. **P < 0.01, unpaired Student’s t test. **(C, D)** Representative images and quantitative analysis of tumor spheres generated by ALDH^+^ and ALDH^-^ (ALDH^low^) HNSCC cells following transfection with the specified siRNAs. Scale bar: 100 μm. Mean ± SD are shown. **p < 0.01, unpaired Student’s t-test. **(E, F)** Limiting dilution analysis showing reduced tumor-initiating capacity of ALDH^+^ CSC-like HN6 cells after LUCAT1 KD (n = 5). Scale bar: 1 cm. Data were processed via Extreme Limiting Dilution Analysis (ELDA) software.

To investigate whether LUCAT1 KD inhibits tumor cell stemness in HNSCC. The ALDEFLUOR assay kit was used to identify CSC-like cells, a method validated in previous studies (13, 15). FACS demonstrated a notable decrease in the ALDH^+^ (ALDH^high^) CSC-like cell population within both HN6 and CAL27 cell lines following LUCAT1 KD (**Figures 2A, B**). The capacity to form tumor spheres represents a standard *in vitro* measure for assessing cancer stem cell properties (13, 16). LUCAT1 knockdown impaired their tumorsphere-forming capacity, reflecting a loss of self-renewal ability (**Figures 2C, D**). Consistently, limiting dilution assays *in vivo* demonstrated that LUCAT1 KD impaired the tumorigenic potential of ALDH^+^ HN6 cells (**Figures 2E, F**). Moreover, LUCAT1 KD suppressed CSC-associated genes (including MYC, ALDH1, BMI1, CD24, OCT4, and SOX2) in ALDH^+^ HN6 and CAL27 cells (**Supplementary Figure 2A**).

### 3.3 LUCAT1 modulates cancer stemness by sponging miR-128

To further elucidate the molecular pathway through which LUCAT1 modulates stemness in HNSCC cells, the subcellular distribution of LUCAT1 was assessed via nuclear-cytoplasmic fractionation assay. Findings indicated that LUCAT1 is primarily localized in the cytoplasm (**Supplementary Figure 3A**). Considering the cytoplasmic localization of LUCAT1 and the well-documented role of miRNAs as key mediators in multiple CSC-related signaling pathways (17, 18), we hypothesized that LUCAT1 regulates cancer stemness by sponging miRNAs.

**Figure 3:**
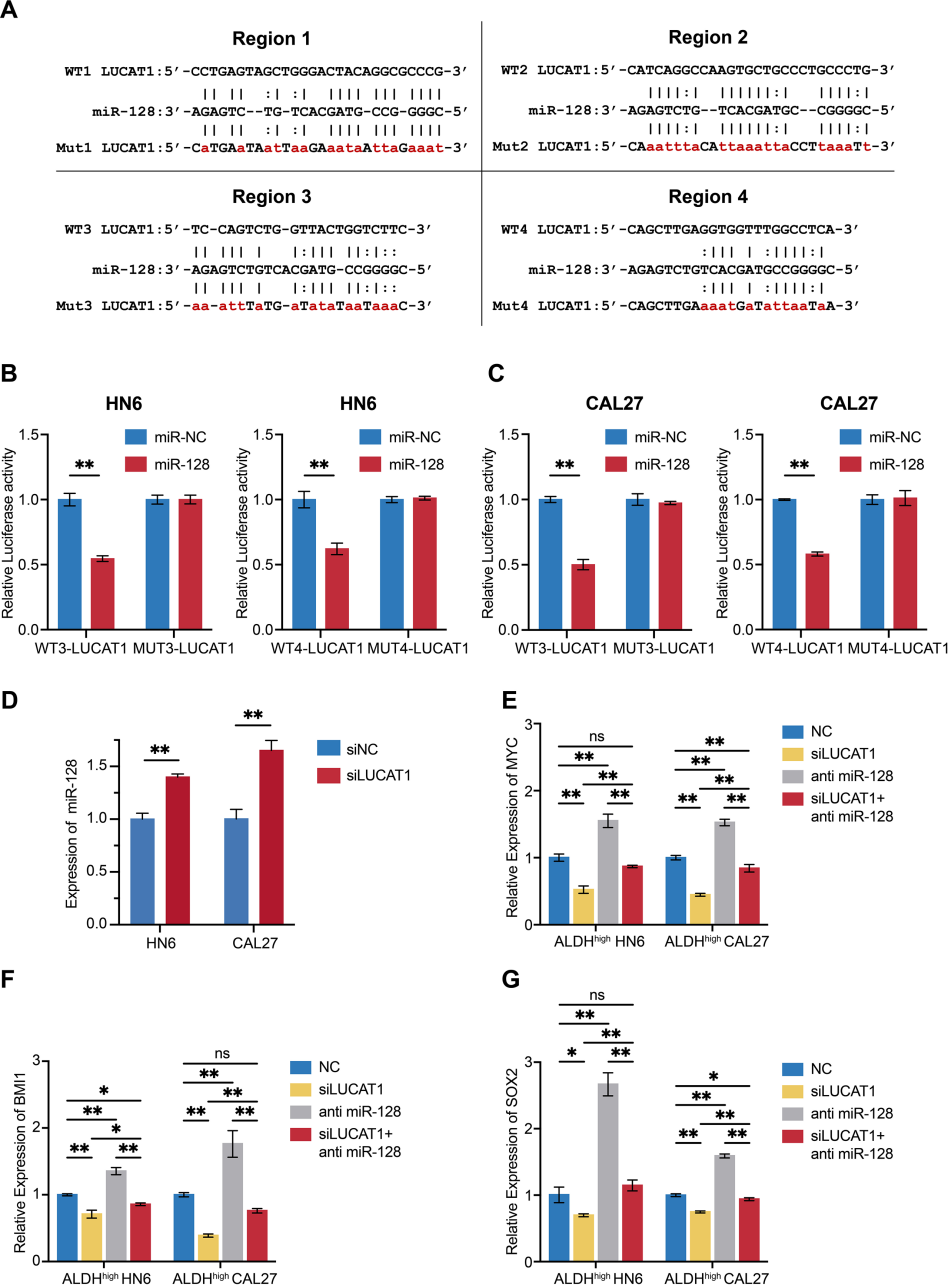
LUCAT1 functions as a miRNA sponge for miR-128. **(A)** Bioinformatic prediction of miR-128 binding sites in LUCAT1. The seed sequence of the miR-128 (middle) matches the wild-type (WT) LUCAT1 binding sites (top), with mutant (Mut) sites shown below. **(B)** Relative luciferase activity in HN6 cells following co−transfection with miR-128 mimic and psiCHECK vectors carrying WT3-LUCAT1, MUT3-LUCAT1, WT4-LUCAT1, or MUT4-LUCAT1. Mean ± SD are shown. **p < 0.01, unpaired Student’s t-test. **(C)** Relative luciferase activity in CAL27 cells following co−transfection with miR-128 mimic and the indicated psiCHECK vectors. Mean ± SD are shown. **p < 0.01, unpaired Student’s t-test. **(D)** miR-128 expression in HN6 and CAL27 cells with LUCAT1 KD, detected by qRT-PCR. Mean ± SD are shown. **p < 0.01, unpaired Student’s t-test. **(E–G)** Expression of stemness-related markers in ALDH^high^ HN6 and CAL27 cells from four groups (negative control, siLUCAT1, miR-128 antagomir, siLUCAT1+miR-128 antagomir), detected by qRT-PCR. Mean ± SD are shown. **p < 0.01, *p < 0.05; ns, no significance (*p* > 0.05), one-way ANOVA.

Among the 579 miRNAs predicted by RNA22 to potentially interact with LUCAT1, candidates were prioritized based on their predicted folding energy. Further filtering using structural parameters and documented tumor−associated functions narrowed the list to four candidates: miR−128, miR−150, miR−566, and miR–665. Subsequent preliminary binding assays revealed that only miR-128 exhibited a robust interaction with LUCAT1. To formally confirm this target relationship, luciferase reporter vectors harboring the predicted miR-128 binding sites of LUCAT1 were constructed (**Figure 3A**). Exogenous miR-128 overexpression significantly inhibited the luciferase activity of reporter plasmids carrying wild-type (WT) LUCAT1 binding sequences (region 3 or region 4), whereas no such inhibition was observed in plasmids with mutant (Mut) sequences (**Figures 3B, C**; **Supplementary Figures 3B, C**). Moreover, LUCAT1 KD significantly increased miR-128 expression in both HN6 and CAL27 cells (**Figure 3D**).

*In vitro* rescue assays showed that treatment with a miR-128 antagomir upregulated the expression of CSCs-related markers at the mRNA levels in ALDH^high^ HN6 and CAL27 cells. Moreover, miR-128 inhibition reversed the inhibitory effect of LUCAT1 KD on cancer stemness (**Figures 3E–G**).

### 3.4 Manipulating miR-128 expression counteracts the oncogenic effects of LUCAT1 *in vitro* and *in vivo*

**Figure 4:**
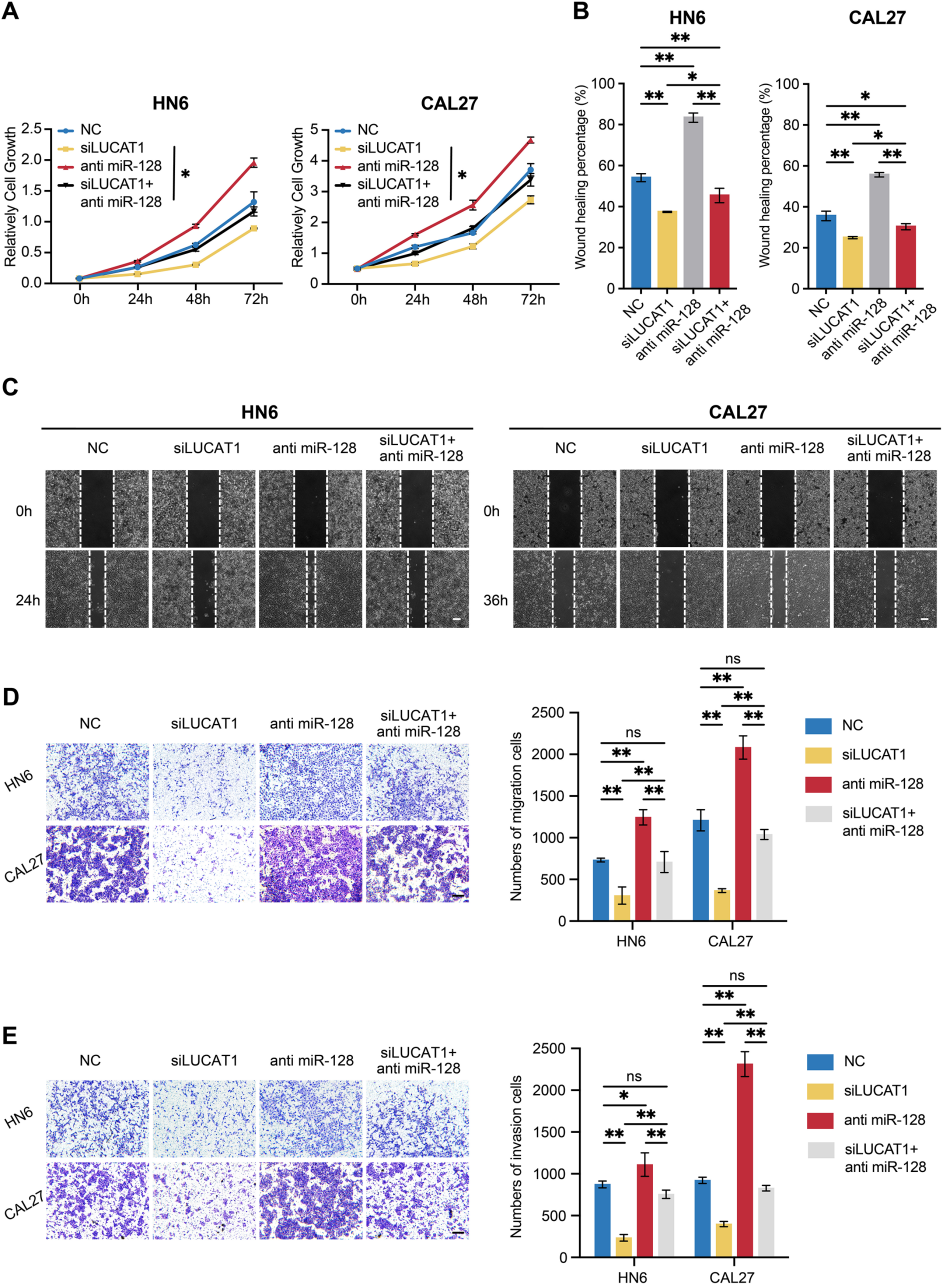
Anti miR-128 attenuates the effects of LUCAT1 knockdown in HNSCC proliferation, invasion, and migration. **(A)** HN6 and CAL27 cells proliferation in the four treatment groups as indicated, assessed by CCK-8 assay. Mean ± SD are shown. *p < 0.05, one-way ANOVA. **(B, C)** Wound healing assays showed that miR-128 antagomir partially reversed the migration inhibition induced by LUCAT1 KD in HN6 and CAL27 cells. Scale bar: 200 μm. Mean ± SD are shown. **p < 0.01, *p < 0.05, one-way ANOVA. **(D)** Transwell migration assays showing the reversal effect of siLUCAT1 + miR-128 antagomir co-transfection on HN6 and CAL27 cell migration. Scale bar: 200 μm. Mean ± SD are shown. **p < 0.01, ns = no significance (*p* > 0.05), one-way ANOVA. **(E)** Transwell invasion assays showing the reversal effect of siLUCAT1+miR-128 antagomir co-transfection on HN6 and CAL27 cell invasion. Scale bar: 200 μm. Mean ± SD are shown. **p < 0.01, *p < 0.05; ns, no significance (p > 0.05), one-way ANOVA.

HN6 and CAL27 cells were transfected with negative control, LUCAT1 KD (siLUCAT1), miR-128 antagomir, or a combination of siLUCAT1 and miR-128 antagomir. The inhibition effects of the miR-128 antagomir were tested (**Supplementary Figure 1B**). Although LUCAT1 KD inhibited proliferation in HN6 and CAL27 cells, this inhibitory effect was reversed by miR-128 inhibition (**Figure 4A**). In line with the proliferation rescue phenotype, the miR-128 antagomir also abrogated the suppressive effects of LUCAT1 KD on the migratory and invasive capacities of HNSCC cells (**Figures 4B–E**). These results demonstrated that the oncogenic activity of LUCAT1 in promoting HNSCC progression is partially mediated by miR-128. To further validate the functional antagonism between LUCAT1 and miR-128, converse rescue experiments were performed by introducing miR-128 mimics into LUCAT1-overexpressing (LUCAT1-OE) HNSCC cells. Functional assays revealed that the enhanced cellular proliferation, migration, and invasion induced by LUCAT1-OE were effectively reversed upon miR-128 upregulation (**Supplementary Figures 4A–C**).

**Figure 5:**
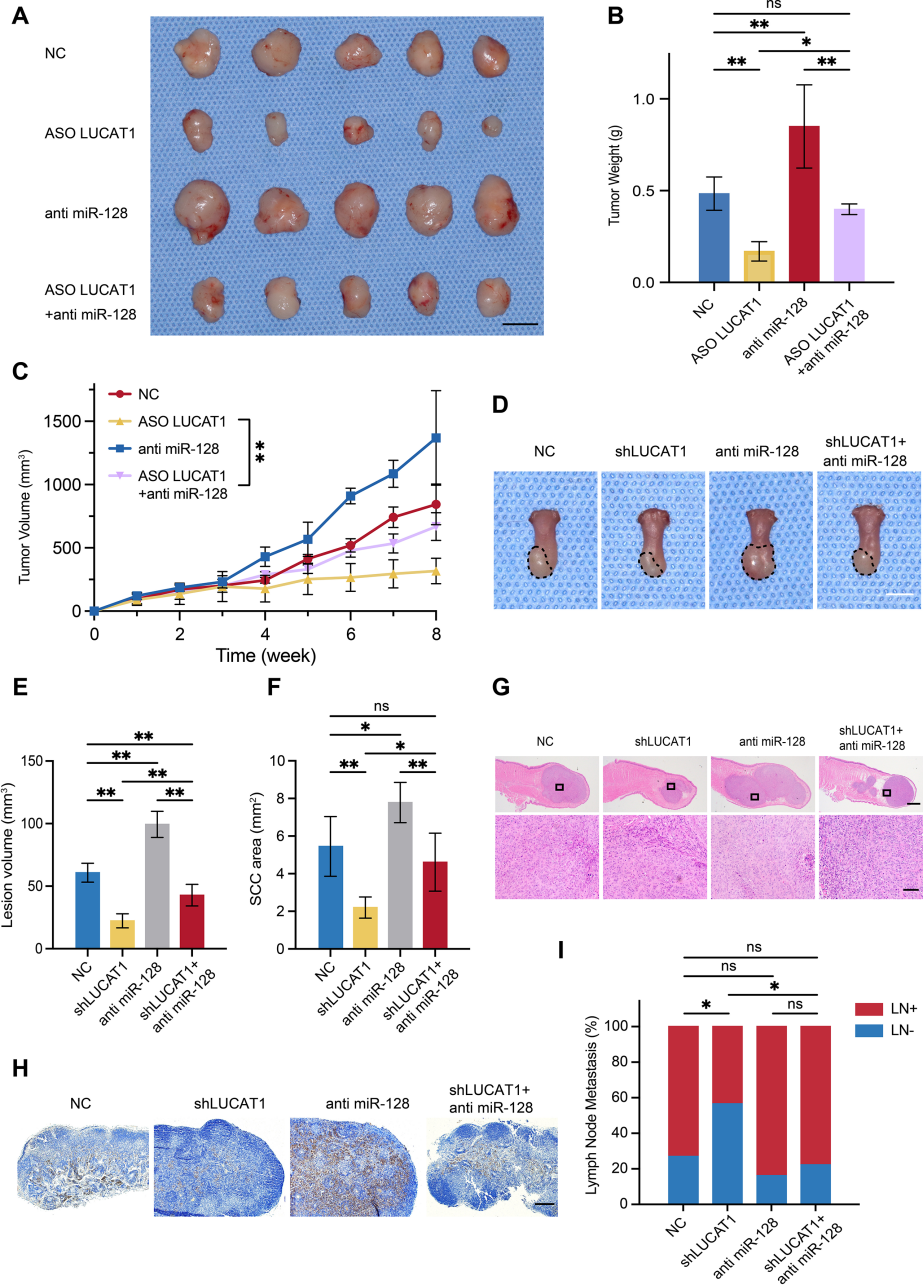
Anti miR-128 partially reverse the anti-tumor effects of LUCAT1 knockdown *in vivo*. **(A)** Representative images of tumors from mice bearing HN6-derived xenografts (NC or shLUCAT1) with or without miR-128 antagomir treatment. Scale bar: 1 cm. **(B)** Tumor weights of subcutaneous xenografts 8 weeks post-treatment. Mean ± SD are shown (n = 5). **p < 0.01, *p < 0.05, ns = no significance (*p* > 0.05), one-way ANOVA. **(C)** The growth curve of subcutaneous xenograft tumor growth in the indicated groups. Mean ± SD are shown (n = 5). *p < 0.05, one-way ANOVA. **(D)** Representative images of orthotopic tongue tumors in mice injected with ALDH^+^ CSC-like HN6 cells, across the indicated treatment groups. Lesion areas are circled with black dotted lines. Scale bar: 5 mm. **(E)** Quantification of orthotopic tumor volume. Mean ± SD are shown (n = 6). **p < 0.01, one-way ANOVA. **(F)** Quantification of the area of the tumors. Mean ± SD are shown (n = 6). **p < 0.01, *p < 0.05, ns = no significance (*p* > 0.05), one-way ANOVA. **(G)** Representative hematoxylin and eosin (H&E) staining of orthotopic tumors. Scale bar, 1 mm; insets (high-magnification views): 100 μm. **(H)** Representative PCK immunohistochemical staining of cervical lymph nodes with cancer metastasis. Scale bar: 200 μm. **(I)** Proportion of metastatic lymph nodes. *p < 0.05; ns, no significance (*p* > 0.05), chi-square test.

Antisense oligonucleotides (ASOs) have been shown to effectively inhibit target genes and significantly inhibit tumor progression *in vivo*, highlighting their potential clinical application for HNSCC treatment (19, 20). LUCAT1 inhibition via ASO significantly inhibited HNSCC tumor growth and tumor volume in nude mice, whereas miR-128 inhibition promoted tumor formation relative to the negative control group (**Figures 5A–C**).

To further explore this rescue effect on lymph node metastasis—a critical determinant of HNSCC prognosis—an orthotopic tongue tumor model was established using FACS-sorted ALDH^+^ CSCs. Consistent with the observed inhibition of tumor growth, LUCAT1 KD suppressed both tumor formation (**Figures 5D–G**) and, more importantly, lymph node metastasis in the orthotopic tongue tumor model (**Figures 5H, I**). Conversely, miR-128 inhibition exerted a potent pro-oncogenic effect, leading to accelerated tumor progression, and its suppression effectively reversed the anti-tumor efficacy induced by LUCAT1 knockdown (**Figures 5D–I**). Quantitative analysis of lymph node metastasis incidence revealed that the control (NC) group exhibited a metastasis rate of 73.1% (19/26). LUCAT1 KD significantly reduced this rate to 43.5% (10/23), whereas miR-128 inhibition alone increased it to 83.9% (26/31). In the combined treatment group (LUCAT1 KD plus anti-miR-128), the metastasis rate was 77.8% (21/27), further confirming that miR-128 inhibition partially rescues the anti-metastatic effect of LUCAT1 KD (**Figure 5I**).

### 3.5 BMI1 is a direct and functional target of miR-128 in HNSCC CSCs

**Figure 6:**
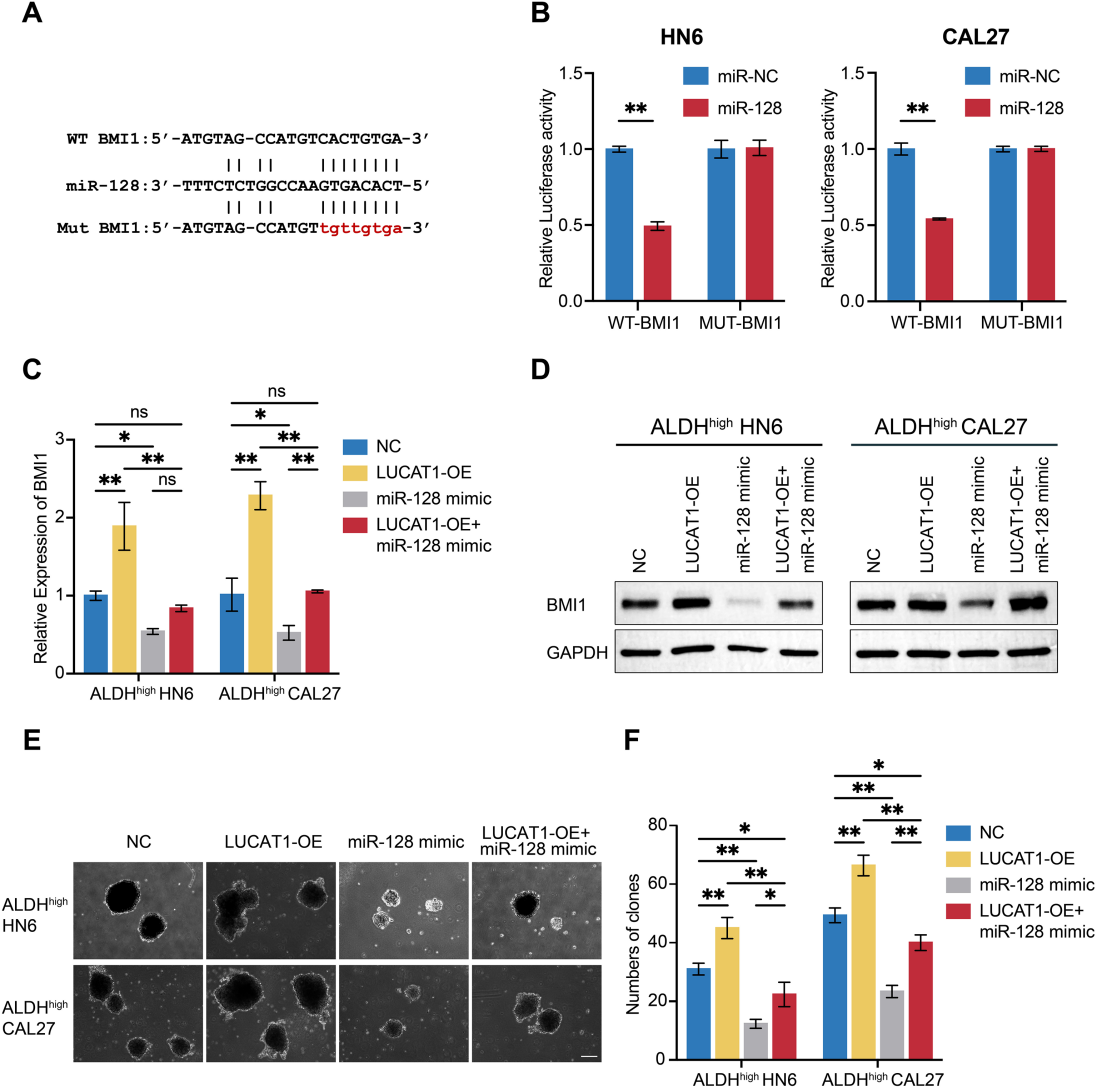
miR-128 functions by directly targeting BMI1 in HNSCC CSCs. **(A)** Representative matched nucleotide sequences between the seed region of miR-128 and the 3’UTR of BMI1. **(B)** Relative luciferase activity in HN6 and CAL27 cells following co−transfection with miR-128 mimic and psiCHECK vectors carrying WT-BMI1, MUT-BMI1. Mean ± SD are shown. **p < 0.01, unpaired Student’s t-test. **(C)** Expression of BMI1 in ALDH^high^ HN6 and CAL27 cells from four groups (negative control, LUCAT1-OE, miR-128 mimic, LUCAT1-OE+miR-128 mimic), detected by qRT-PCR. Mean ± SD are shown. **p < 0.01, *p < 0.05, ns = no significance (*p* > 0.05), one-way ANOVA. **(D)** Western blot analysis of BMI1 in ALDH^+^ HN6 and CAL27 cells following indicated treatments. GAPDH was used as the internal control. **(E, F)** Representative images and quantitative analysis of tumor spheres generated by ALDH^high^ HNSCC cells following indicated treatments. Scale bar: 100 μm. Mean ± SD are shown. **p < 0.01, *p < 0.05, unpaired Student’s t-test.

To elucidate the downstream mechanism by which miR-128 mediates LUCAT1-induced stemness, we focused on BMI1, a well-known stemness-related oncogene and a predicted target of miR-128 (21, 22). Based on previous studies (22), we constructed luciferase reporter plasmids harboring a fragment of the BMI1 sequence that contains the putative miR-128 binding sites (**Figure 6A**). In HN6 and CAL27 cells, the luciferase activity of the plasmid with the wild-type (WT) miR-128 binding sites was effectively suppressed by miR-128, whereas the activity of the plasmid with the mutant (Mut) sequences was not affected (**Figure 6B**).

Following the confirmation of LUCAT1 overexpression efficiency (**Supplementary Figure 1C**), we assessed BMI1 expression at both the transcript and protein levels. As expected, LUCAT1-OE significantly upregulated BMI1, whereas the introduction of miR-128 mimics decreased its expression. Importantly, co-transfection assays revealed that miR-128 could partially reverse the LUCAT1-induced upregulation of BMI1 (**Figures 6C, D**). To further evaluate the biological impact of this LUCAT1/miR-128/BMI1 regulatory axis on cancer stemness, we performed tumor sphere formation assays using ALDH^high^ HNSCC cells. Consistent with the molecular alterations, miR-128 functionally counteracted the LUCAT1-driven enhancement in stemness, as evidenced by significant reductions in both the number and size of the formed tumor spheres (**Figures 6E, F**).

## 4 Discussion

As a conserved lncRNA, LUCAT1 emerges as an oncogenic in modulating tumor initiation and progression in various tumors (7). Prior studies, including our recent clinical observations, have demonstrated that elevated LUCAT1 expression correlates with poor prognosis in HNSCC patients (23). In line with these clinical findings, our current data firmly establish its oncogenic role in driving HNSCC cell proliferation, migration, and invasion. Moreover, we uncover a robust link between LUCAT1 and HNSCC cancer stem cells (CSCs): LUCAT1 silencing not only diminished the fraction of CSCs within HNSCC cell populations but also compromised their self-renewal potential and *in vivo* tumorigenic capacity, highlighting LUCAT1 as a critical regulator of cancer stemness in HNSCC.

LUCAT1 exerts context-dependent oncogenic effects, reflecting its functional versatility. For instance, exosome−transmitted LUCAT1 binds IGF2BP2 in an m6A−dependent fashion to stabilize HMGA1 mRNA, amplifying oncogenic signaling cascades (24). Moreover, LUCAT1 interacts with PTBP1 to recruit DNA damage−related transcripts and alter their alternative splicing, contributing to genomic instability (25). It also facilitates tumorigenesis and metastasis by sequestering miR-5702 directly (26) or acting as a molecular sponge for miR-4316 to upregulate VEGFA, driving metabolic reprogramming and pro-metastatic programs (27). In contrast to these known modes of action, our study focuses on LUCAT1’s role in competitive endogenous RNA (ceRNA) networks—specifically miRNAs regulating CSC stemness—uncovering a previously unrecognized regulatory axis in HNSCC.

The subcellular localization of lncRNAs defines their functional roles: nuclear lncRNAs typically regulate gene expression via epigenetic or transcriptional mechanisms, while cytoplasmic lncRNAs mediate post-transcriptional and translational processes (7). LUCAT1 cytoplasmic localization led us to hypothesize a role in post-transcriptional regulation. Bioinformatic analysis coupled with luciferase reporter experiments verified that miR−128 is a direct binding target of LUCAT1. Functionally, miR-128 inhibition partially reversed the anti-stemness and anti-tumor effects of LUCAT1 KD *in vitro* and *in vivo*, establishing miR-128 as a critical downstream mediator of LUCAT1.

The miR-128 is well established as a tumor suppressor targeting multiple oncogenic pathways (28, 29). Our study extends these results by demonstrating that LUCAT1 acts as a molecular sponge to sequester miR-128, thereby derepressing its downstream target BMI1, sustaining the CSCs phenotype, and driving HNSCC progression. Evidence has shown that miR-128 exerts multi-faceted tumor-suppressive effects across cancers: it inhibits the PRKCQ axis in colorectal cancer (29), suppresses CDC6-driven proliferation and survival in hepatocellular carcinoma (30), and blocks epithelial-to-mesenchymal transition (EMT) and metastasis in lung cancer through the RhoA pathway (31).

Progress in RNA synthesis, modification, and delivery technologies has accelerated the development of RNA-based therapeutics (32), offering precise, versatile tools to regulate gene expression and revolutionize the treatment of genetic diseases and cancer (33). Antisense oligonucleotides (ASOs) are short, synthetic nucleic acids that bind target RNA, mainly via an RNase H-dependent mechanism, to trigger enzymatic degradation of the target and reduce downstream protein levels (34). Several ASOs have been approved for clinical application (33); for instance, LUCAT1-targeting ASOs combined with oxaliplatin exhibit potent therapeutic effects by inhibiting tumor growth in PTBP1-driven hypoxic colorectal cancer models (25). Our *in vivo* data show that LUCAT1 inhibition via ASOs attenuates HNSCC tumor growth. Given the correlation between elevated LUCAT1 expression and poor clinical outcomes in HNSCC patients, therapeutic strategies targeting the LUCAT1/miR-128 axis show considerable promise for HNSCC therapy.

Despite the functional and mechanistic insights provided by our *in vitro* and *in vivo* results, several limitations in the current study should be acknowledged. Although our previous work (23) demonstrated that elevated LUCAT1 expression correlates with advanced tumor stage, lymph node metastasis, and poor prognosis in HNSCC patients, the clinical expression levels of miR-128 and BMI1, as well as the clinical relevance of the complete LUCAT1/miR-128/BMI1 regulatory axis, remain to be fully validated in patient samples. Therefore, future studies using independent clinical cohorts or public databases are warranted to verify the combined expression patterns of these three molecules.

In summary, our results demonstrated that LUCAT1 was a potential therapeutic target for HNSCC by regulating CSC stemness, with miR-128 acting as a key intermediate mediator and BMI1 as the direct downstream target. This study shows a novel LUCAT1/miR-128/BMI1 ceRNA regulatory axis in governing HNSCC CSC properties, laying a foundation for the development of anti-CSCs targeted therapies against this malignancy.

## Data Availability

The original contributions presented in the study are included in the article/**Supplementary Material**. Further inquiries can be directed to the corresponding authors.
