# Supplementary material for: LUCAT1 promotes stemness in head and neck squamous cell carcinoma by sponging miR-128
Source: Front Oncol. 2026 Jun 3;16:1795813. doi: 10.3389/fonc.2026.1795813 (PMC13272036; doi:10.3389/fonc.2026.1795813)
Supplement: Supplementary file 1 [file DataSheet1.docx]

Supplementary Material

# Supplementary Figures and Tables

## Supplementary Figures


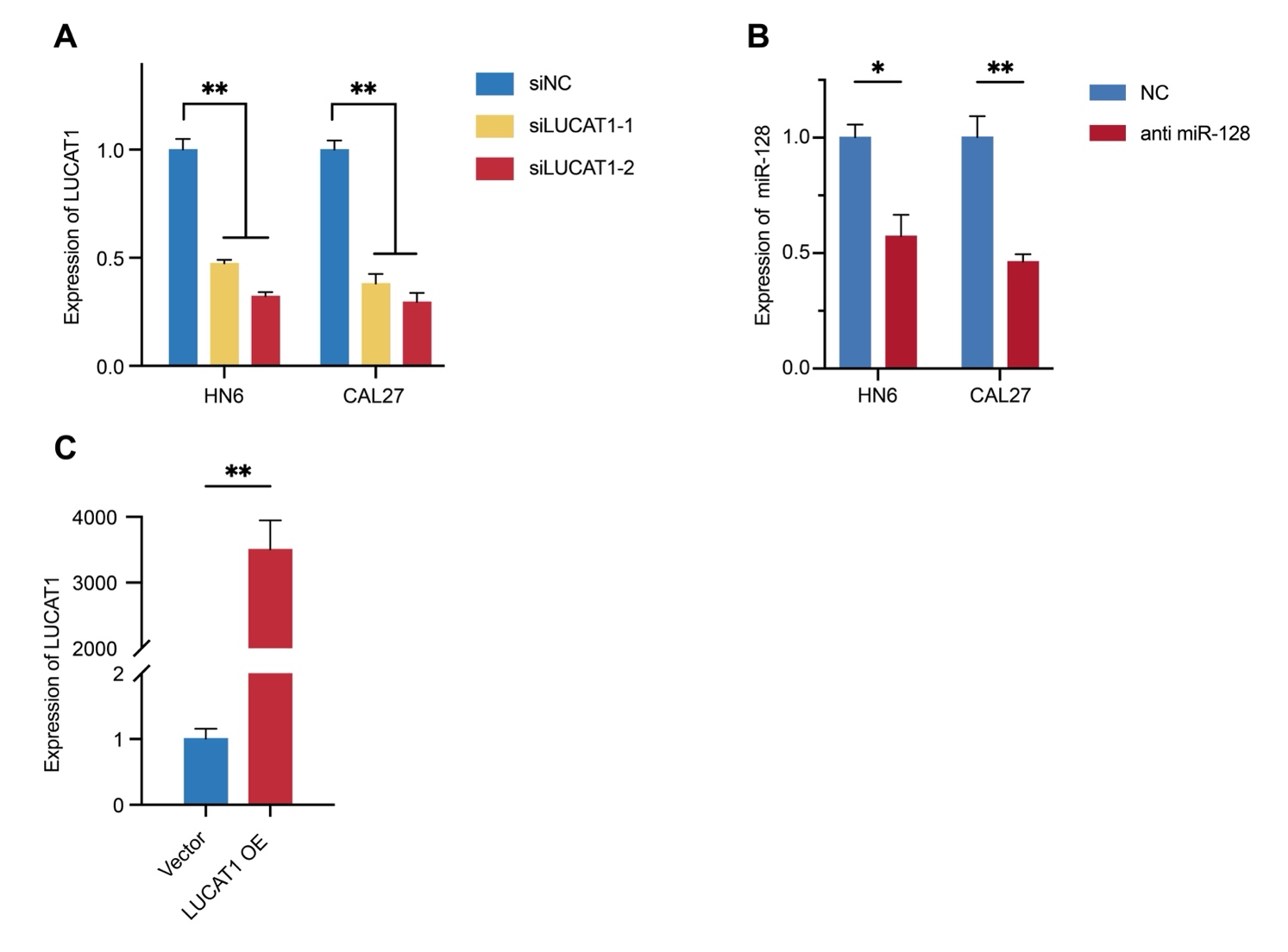


**Supplementary Figure S1.** **Genetic manipulation. (A)** LUCAT1 expression in HN6 and CAL27 cells transfected with siNC, siLUCAT1-1, or siLUCAT1-2 for 48 h, detected by qRT-PCR. Mean ± SD are shown. **p < 0.01, unpaired Student’s t-test. **(B)** miR-128 expression in HN6 and CAL27 cells transfected with miR-128 antagomir, detected by qRT-PCR. Mean ± SD are shown. **p < 0.01, *p < 0.05, unpaired Student’s t-test. **(C)** LUCAT1 expression in HN6 cells transduced with the vector and LUCAT1-OE plasmid. Data are shown as the mean ± SD. ***p* < 0.01 using an unpaired Student’s t-test.


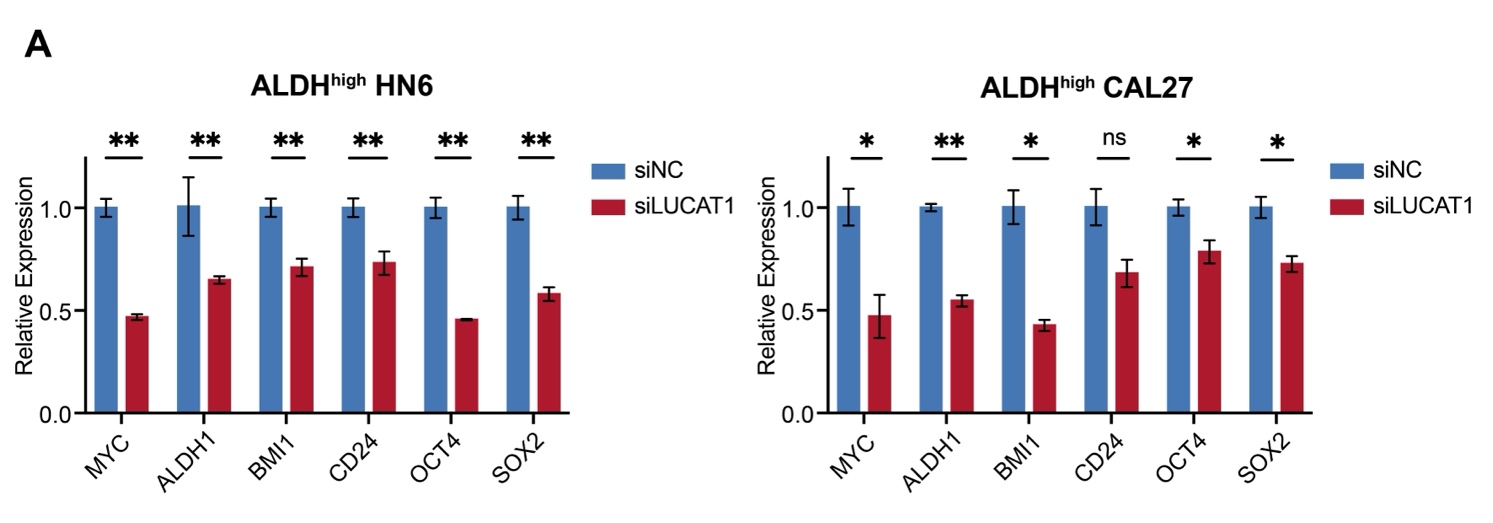


**Supplementary Figure S2.** **LUCAT1 down-regulation inhibits the HNSCC stem cell marker expression. (A)** Stemness-related markers’ expression in ALDH⁺ HN6 and CAL27 cells following LUCAT1 KD, detected by qRT-PCR. Mean ± SD are shown. ***p* < 0.01, **p* < 0.05, ns = no significance (p > 0.05), unpaired Student’s t-test.


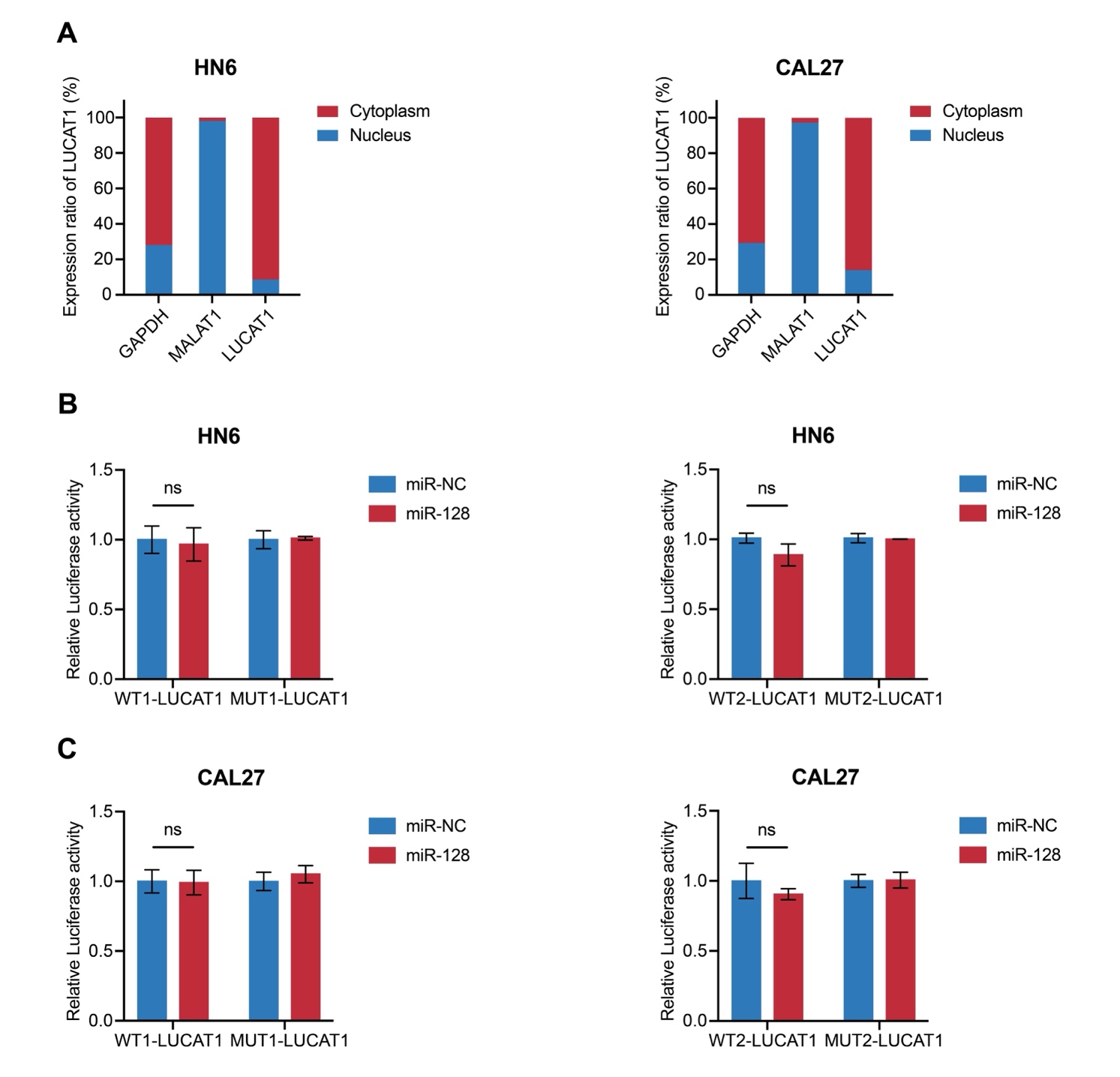


**Supplementary Figure S3.** **LUCAT1 functions as a sponge for miR-128. (A)** Subcellular localization of LUCAT1 in HN6 and CAL27 cells, detected by qRT-PCR. MALAT1 and GAPDH served as nuclear and cytoplasmic controls, respectively. **(B)** Relative luciferase activity in HN6 cells co-transfected with miR-128 mimic and psiCHECK vectors carrying WT1-LUCAT1, MUT1-LUCAT1, WT2-LUCAT1, or MUT2-LUCAT1. Mean ± SD are shown. **p < 0.01, unpaired Student’s t-test. **(C)** Relative luciferase activity in CAL27 cells co-transfected with miR-128 mimic and the indicated psiCHECK vectors. Mean ± SD are shown. **p < 0.01, unpaired Student’s t-test.


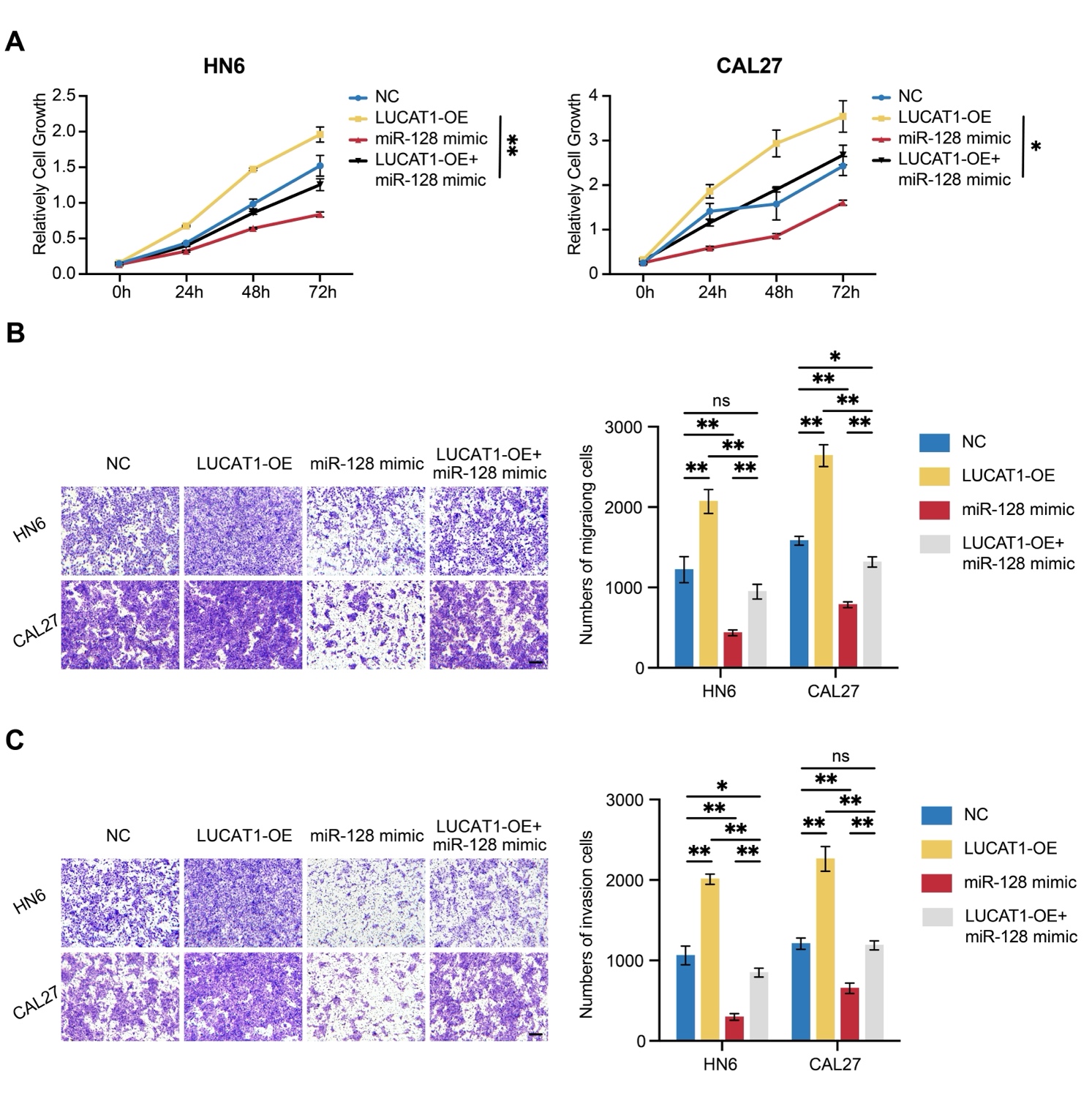


**Supplementary Figure S4.** **miR-128 mimics attenuate the effects of LUCAT1 overexpression in HNSCC proliferation, invasion, and migration. (A)** HN6 and CAL27 cells proliferation in the four treatment groups as indicated, assessed by CCK-8 assay. Mean ± SD are shown. ***p* < 0.01, **p* < 0.05, one-way ANOVA. **(B)** Transwell migration assays showing the reversal effect of LUCAT1-OE + miR-128 mimics co-transfection on HN6 and CAL27 cell migration. Scale bar: 200 μm. Mean ± SD are shown. **p < 0.01, *p < 0.05, ns = no significance (p > 0.05), one-way ANOVA. **(C)** Transwell invasion assays showing the reversal effect of LUCAT1-OE + miR-128 mimics co-transfection on HN6 and CAL27 cell invasion. Scale bar: 200 μm. Mean ± SD are shown. **p < 0.01, *p < 0.05, ns = no significance (p > 0.05), one-way ANOVA.

## Supplementary Tables

**Table S1.** Sequences of RNA

|  | Sequence (5′-3′) |
| --- | --- |
| siLUCAT1-1 | GCUCCUUUCCUCACAAGAA |
| siLUCAT1-2 | CACACUAUGUGUUCUGACU |
| shLUCAT1 | CCTTAGACAGGTGCAATTT |
| ASO NC | CCTTCCCTGAAGGTTCCTCC |
| ASO LUCAT1 | GCCTGTACAGTTGTGTCCAA |
| Antagomir miR-128 | CGGGGCCGUAGCACUGUCUGAGA |
| miR-128 mimics | CGGGGCCGUAGCACUGUCUGAGA |

**Table S2.** Sequences of qRT-PCR primers.

| Target genes | Forward (5′-3′) | Reverse (5′-3′) |
| --- | --- | --- |
| GAPDH | GGAGCGAGATCCCTCCAAAAT | GGCTGTTGTCATACTTCTCATGG |
| LUCAT1 | TTGGCACCAGAGACCACAAA | GGGCGACAGAGCGAAACTCT |
| MALAT1 | AAAGCAAGGTCTCCCCACAAG | GGTCTGTGCTAGATCAAAAGGCA |
| MYC | GTCAAGAGGCGAACACACAAC | TTGGACGGACAGGATGTATGC |
| BMI1 | CGTGTATTGTTCGTTACCTGGA | TTCAGTAGTGGTCTGGTCTTGT |
| CD24 | TGAAGAACATGTGAGAGGTTTGAC | GAAAACTGAATCTCCATTCCACAA |
| SOX2 | GCCGAGTGGAAACTTTTGTCG | GGCAGCGTGTACTTATCCTTCT |
| ALDH1 | GCACGCCAGACTTACCTGTC | CCTCCTCAGTTGCAGGATTAAAG |
| OCT4 | TCCCATGCATTCAAACTGAGG | CCTTTGTGTTCCCAATTCCTTCC |
| U6-qPCR | CTCGCTTCGGCAGCACA | AACGCTTCACGAATTTGCGT |
| U6-RT | AACGCTTCACGAATTTGCGT | |
| miR-128-qPCR | AATAGTCGGGGCCGTAGCACT | ATCCAGTGCAGGGTCCGAGG |
| miR-128-RT | GTCGTATCCAGTGCAGGGTCCGAGGTATTCGCACTGGATACGACTCTCAG | |
